# Cholera hotspots and surveillance constraints contributing to recurrent epidemics in Tanzania

**DOI:** 10.1186/s13104-019-4731-0

**Published:** 2019-10-21

**Authors:** Yaovi M. G. Hounmanou, Kåre Mølbak, Jonas Kähler, Robinson H. Mdegela, John E. Olsen, Anders Dalsgaard

**Affiliations:** 1 Department of Veterinary and Animal Sciences, Faculty of Health and Medical Sciences, University of Copenhagen, 1870 Frederiksberg C, Copenhagen, Denmark; 2 Division of Infectious Disease Preparedness, Statens Serum Institut, Artillerivej 5, 2300 Copenhagen, Denmark; 3 Department of Veterinary Medicine and Public Health, College of Veterinary and Biomedical Sciences, Sokoine University of Agriculture, PO Box: 3021, Morogoro, Tanzania; 4 School of Chemical and Biomedical Engineering, Nanyang Technological University, Singapore, Singapore

**Keywords:** Cholera, Tanzania, Spatial–temporal analysis, Great Lakes, Cholera dynamics

## Abstract

**Objective:**

We described the dynamics of cholera in Tanzania between 2007 and 2017 and assessed the weaknesses of the current surveillance system in providing necessary data in achieving the global roadmap to 2030 for cholera control.

**Results:**

The Poisson-based spatial scan identified cholera hotspots in mainland Tanzania. A zero-inflated Poisson regression investigated the relationship between the incidence of cholera and available demographic, socio-economic and climatic exposure variables. Four cholera hotspots were detected covering 17 regions, home to 28 million people, including the central regions and those surrounding the Lakes Victoria, Tanganyika and Nyaza. The risk of experiencing cholera in these regions was up to 2.9 times higher than elsewhere in the country. Regression analyses revealed that every 100 km of water perimeter in a region increased the cholera incidence by 1.5%. Due to the compilation of surveillance data at regional level rather than at district, we were unable to reliably identify any other significant risk factors and specific hotspots. Cholera high-risk populations in Tanzania include those living near lakes and central regions. Successful surveillance require disaggregated data available weekly and at district levels in order to serve as data for action to support the roadmap for cholera control.

## Introduction

Half of all cholera reported cases from Africa between 1970 and 2011 were notified by seven countries, including Tanzania, which has remained one of the top cholera reporting countries until 2018 [1, 2]. Since the seventh cholera pandemic reached the country in 1974, Tanzania reports outbreaks almost every year and has notified over 250,000 cases and 13,078 deaths by 2018 [1, 3].

In 2017, the global task force on cholera control established a roadmap to 2030 for elimination of cholera with Tanzania being one of the 48 targeted endemic countries [4]. The strategies recommended by the task force include rigorous surveillance for early detection and response with emphasis on cholera high-risk populations at local levels for optimal interventions [4, 5]. Therefore, prioritizing high-risk areas in endemic countries can increase the efficiency of cholera control programs because only detailed analysis of local data in each country can provide better understanding of local cholera dynamics for effective control [1]. Successful identification of high-risk areas, however depends on robust surveillance, which is difficult to achieve in many countries including Tanzania due to the stigma related to cholera reporting and associated economic losses in the tourism sector leading to inaccurate reporting in many countries [5]. Nevertheless, a number of spatio-temporal studies have been conducted with surveillance data in Uganda, in the Democratic Republic of Congo (DRC), in India and on Zanzibar islands to contribute with scientific knowledge enabling these countries to get rid of epidemic cholera by 2030 [6–10].

In the present study, we analyzed 11 years of available cholera surveillance data between 2007 and 2017 from all regions of mainland Tanzania. Together with geographical, climatic and socio-demographic data, hotspots identification and risk factors analyses were performed to describe the epidemiology of cholera in Tanzania and address weaknesses in the current surveillance system in achieving the objectives of the global roadmap to 2030 for elimination of cholera in the country.

## Main text

### Methods

#### Data collection

Retrospective data of cholera cases and deaths compiled at regional levels from 2007 to 2017 were obtained from the Epidemiology and Disease Control Section of the Tanzanian Ministry of Health. Data included in this study are those reported based on the WHO standards for cholera case definition and are described as follows:

(i) Patient aged 5 years or more with severe dehydration and acute watery diarrhea or individual who died from the same symptoms in an area without a confirmed outbreak;
(ii) A patient aged 2 years or above having acute watery diarrhea, with or without vomiting in an area where there is an ongoing cholera epidemic;
(iii) Confirmation of *Vibrio cholerae* O1 isolated in the stool of suspected patients.

Population data for the country’s 25 regions (Additional file 1) were extracted from the latest 2012 Population and Housing Census report from the National Bureau of Statistics [11]. Using the inter-censual growth rate of each region, annual population in each region was calculated to estimate the population at risk per year in each region. Proportion of households with access to improved drinking water and households with access to improved toilets were retrieved and included in the analysis. For indicators of socioeconomic status, proportion of households possessing mobile phone and television during the census was used (Additional file 1).

Rainfall data were obtained from the Tanzanian Meteorological Agency, where annual rainfall data in millimeter was obtained per region for each of the 11 years.

Country shape files were obtained from the National Bureau of Statistics [11]. GIS data of the water areas were obtained online from diva-GIS website [12]. All shape files were analyzed in quantum GIS version 2.18, Las Palmaras (https://qgis.org/en/site/) for validity. In QGIS, regional and waterbodies polygons were joined using the Union vector to determine two new variables: total water area and water perimeter found in each region and included in the analysis (Additional file 1).

#### Data analyses

Identification of high risk clusters (hotspots) were performed using SaTScan v 9.6 (https://www.satscan.org/) with the Poisson-based spatial scan. The centroid coordinates of each region was detected from the regional shape-file using QGIS 2.18. These coordinates were used as the geographic references of the regions. In the Poisson model, the expected cholera cases in each part of the regions are assumed proportional to the population size of the region. The model detected clusters in a multidimensional point process and allowed variable window sizes to scan for cholera cases within the region. Variable window size was used, because a prior estimation of the size of the area covered by a cluster was not known. A circular scan window was selected, which moved over the entire region with a radius that varied from zero to 25% of the population at risk. The clusters covered areas with lower rates outside a circular scan window compared with higher rates inside the circle. The likelihood ratio for a specific window was determined as previously described [6, 8]. The output files were displayed in Google Map.

**Fig. 1:**
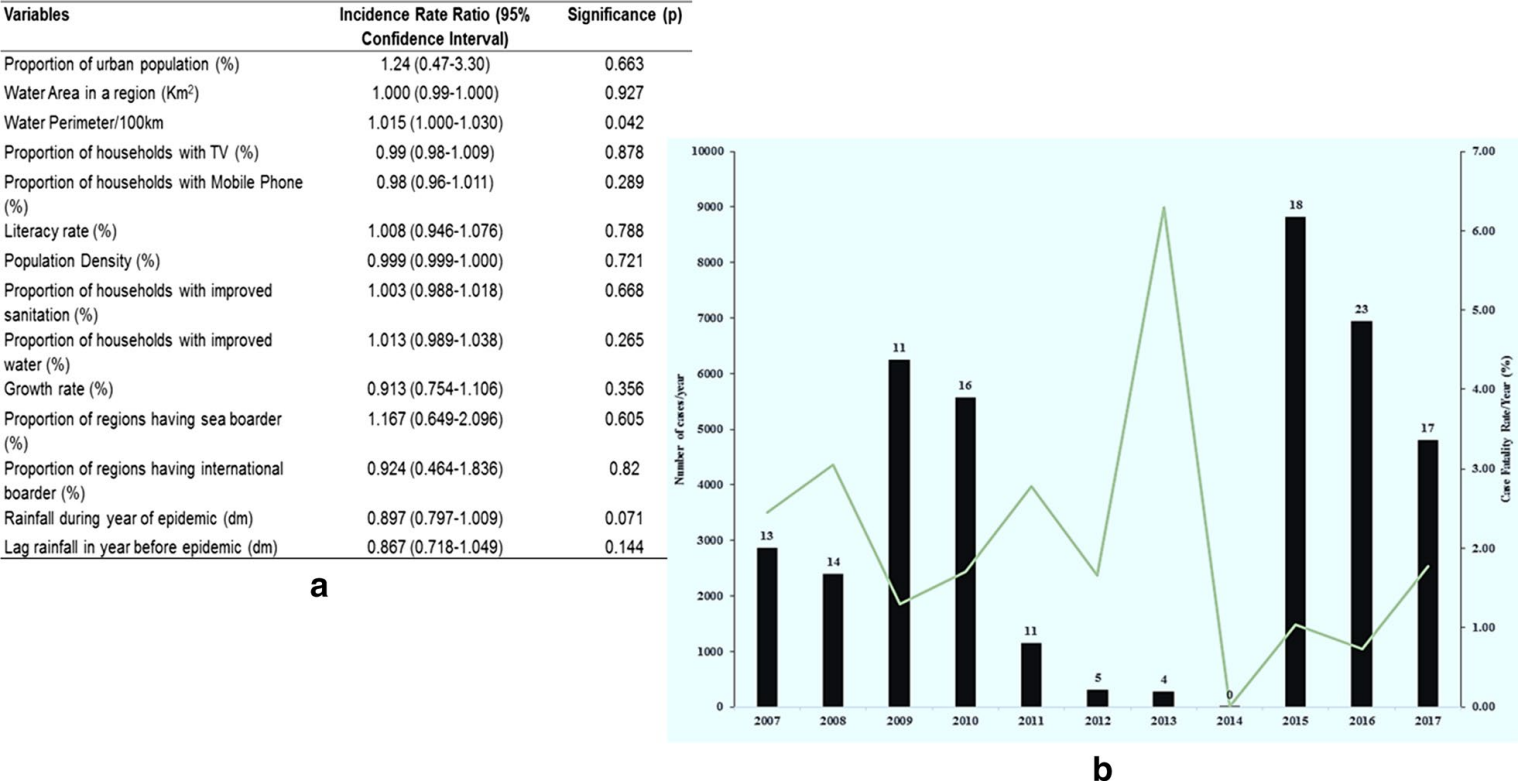
**a** Poisson regression of determinants for cholera reporting in Tanzania based on regional data. **b** Number of cholera cases (bars) and case fatality rates (line) per year in mainland Tanzania. Numbers of affected regions are shown on top of each bar

Poisson regression was used for the analyses of potential risk factors for cholera. In this model, the total number of cases (2007 to 2017) reported at the regional level was the dependent variable, exposure variables were obtained from the 2012 census as well as parameters from the geographical analysis (Fig. 1a). The logarithm to the population was included as an offset variable and thereby the analysis represents a log-linear model of the incidence. We used a zero-inflated Poisson regression for the analysis of the relation between rainfall in the year before reporting and the number of reported cases. In both models, we applied robust standard errors. In the analysis of the relation between rainfall and number of reported cases, we adjusted the standard error for the 25 regions because it was assumed that there is less variance within a region than between regions. The log to the estimated yearly population was included as offset variable. Stata version 14 was used for the regression models.

### Results and discussion

From 2007 to 2017, mainland Tanzania reported 39,444 cholera cases with 600 deaths, giving a case fatality rate of 1.5% and an average annual incidence rate of 8.39 per 100,000 people. A similar analysis of 10 years data from the DRC revealed a higher number of cases and case fatality rates of 1.9% [9]. DRC is the country reporting the most cholera cases in Africa and for many years have been a devastated country because of wars and population displacement associated with higher risks for cholera [9]. However, the relatively lower incidence in Tanzania could be attributed to the fact that reporting cholera has negative impacts on tourism and exports of affected countries, leading to underreporting [5]. In 2016, 23 of the 25 regions in Tanzania reported cholera but most cases (8821) were reported in 2015 (Fig. 1b). The highest case fatality rate was recorded in 2013 (6.3%) when only 270 cases were reported countrywide. It is likely that initial cases in an outbreak experience elevated case-fatality, whereas the official recognition of an outbreak leads to improved management and increased case-finding thereby identifying milder cases as well. At regional level, Shinyanga had the highest case fatality rate in the study period (7%) while Dodoma reported the highest number of cases (5988), although 94.1% of these cases were recorded exclusively between 2015 and 2017 where the countrywide incidence reached 14.2 per 100,000 people (Fig. 1b). Every region except Kagera, reported cholera at least once during the 11 years. Fifteen regions reported cholera in at least six of the 11 years and can be considered cholera endemic regions [6, 8].

**Fig. 2:**
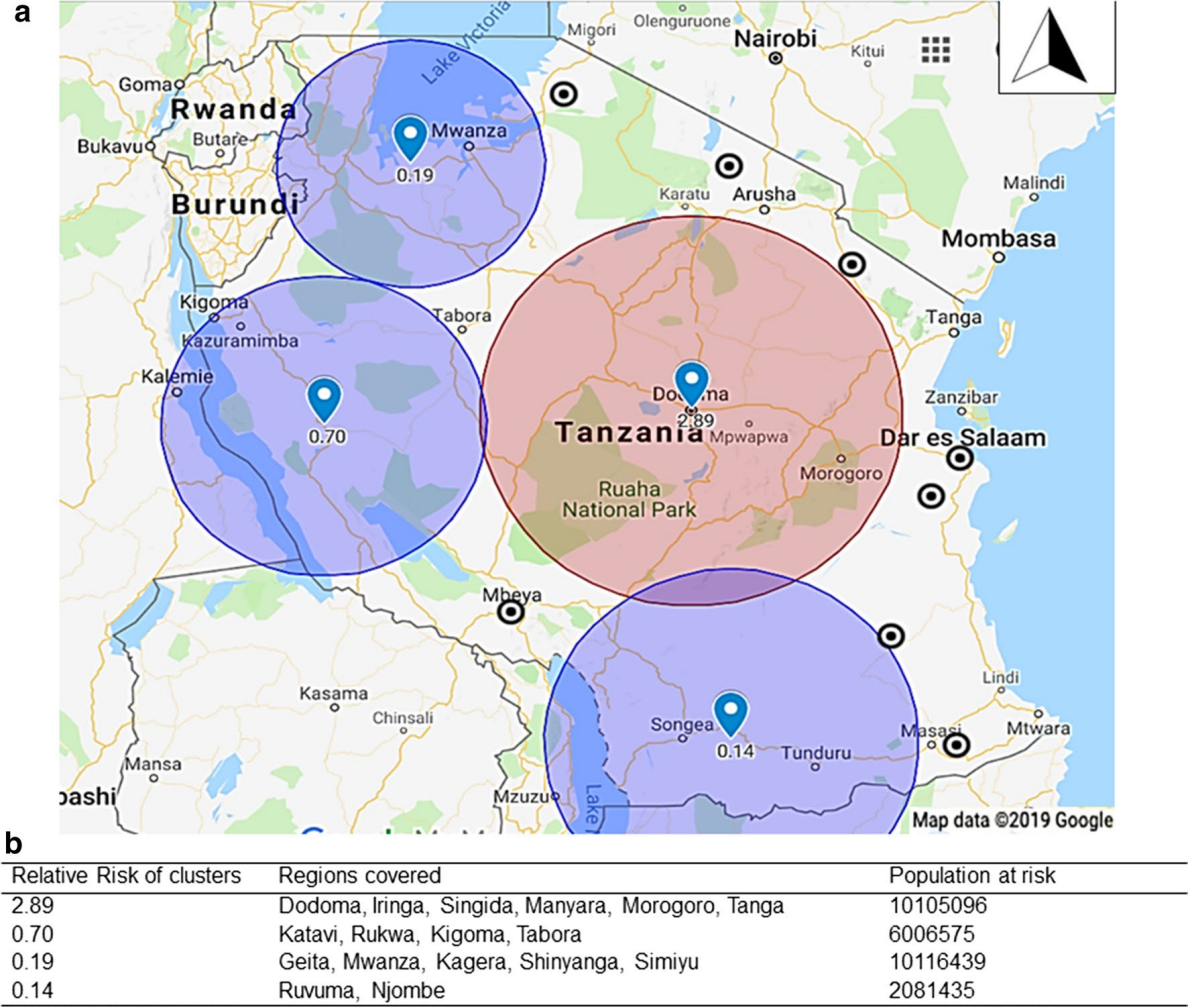
Cholera hotspots in mainland Tanzanian and relative risks as identified by SatScan and displayed using Google map. **a** Mapping of the relative risks of cholera within the identified hotspots. **b** Regions at risk and population size in the identified cholera hotspots

Spatial analyses revealed four high-risk areas of different sizes (Fig. 2a). Seventeen regions had their centroids within the identified hotspots. The risk of having cholera in these regions was up to 2.89 times higher compared to elsewhere in the country (p < 0.0001, Fig. 2b). The hotspot with highest risk of cholera was Dodoma region, including its neighboring regions where high magnitude outbreaks were recorded after 2015. This corroborates the role of urbanization and population displacements in cholera dynamics [13–15]. The increasing number of outbreaks in 2015–2017 in Dodoma coincides with the time where the Tanzanian government moved offices to Dodoma as the capital city of the country. This was associated with significant movement of government employees and affiliated business from Dar es Salaam, but also a number of people working with the construction of new government office buildings [16].

Approximately 28 million people live in the regions found in the hotspots based on the 2012 census. Three of the four hotspots were around major lakes in the country mainly Lake Victoria, Tanganyika and Nyasa. Living near a lake was also reported in Uganda, the DRC and elsewhere as a factor associated with increased cholera incidence [8, 17, 18].

According to existing literature, Dar es Salaam is one of the endemic cities experiencing cholera outbreaks in Tanzania [19]. This city was however not identified in our analysis as part of the hotspot areas. Such a discrepancy reveal one of the limitations of the data and weaknesses of the existing surveillance system in which cholera reports were aggregated at regional level rather than at district level. The observed clusters should therefore be treated with caution. Reliable disease hotspot identification cannot be effective when surveillance and risk factor data are not available from the smallest geographical structures in affected countries [6, 8]. Reliability of data also includes consistency in case definitions because only a small proportion of suspected cases are laboratory confirmed and may bias identification of priority areas where interventions are needed [5].

**Fig. 3:**
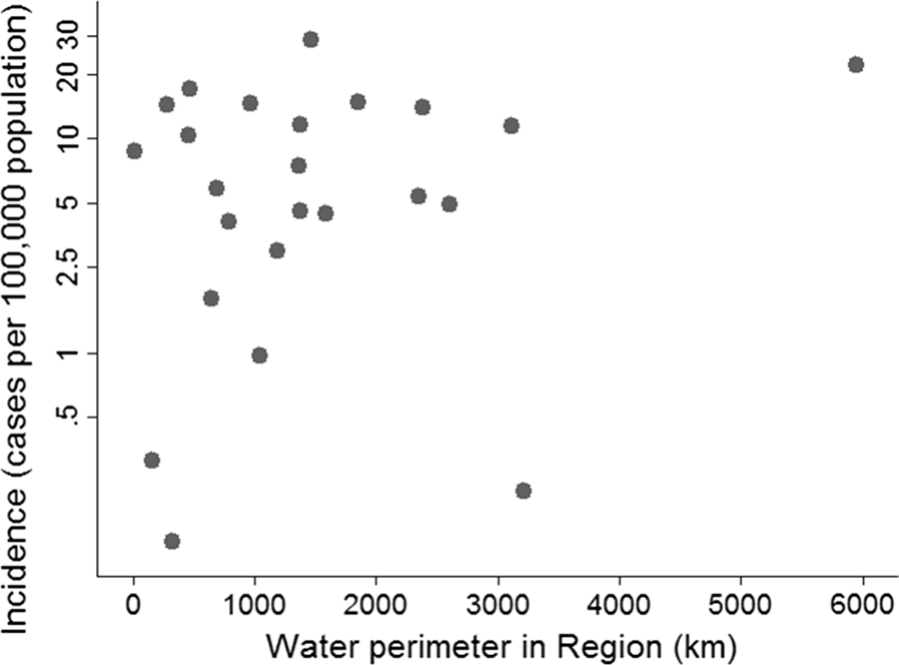
Relationship between cholera incidence (log incidence per 100,000 population) and total water perimeter (in kilometer) in a region. One region has been omitted, i.e. Kagera because of zero reporting

Compared to previous studies in the DRC, Uganda, India and Zanzibar [6–9] which had cholera data and exposure variables from districts, the available regional data in Tanzania were not able to detect many risk factors investigated against the incidence of cholera. The rainfall pattern, both before and during the year of epidemics did not significantly affect the cholera incidence in mainland Tanzania based on currently available data (p = 0.07; 0.14, respectively, Additional file 1). This could mean that in reality there is no positive correlation between rainfall and risk for cholera in mainland Tanzania. Nevertheless, cholera is normally expected to have a seasonal pattern as is the case in Zanzibar and elsewhere [7, 20, 21]. Only water perimeter in a region was significantly associated with incidence of cholera with a 1.5% increase in incidence for 100 km increase in water perimeter (IRR 1.015; 95% CI 1.001 to 1.030; p = 0.042, Fig. 3). This correlates findings of the hotspots analyses where three significant clusters covered regions around the Great Lakes. Living near water bodies mainly great Lakes seems therefore a significant risk factor for cholera which has also been documented globally [22]. Recent findings in Tanzania where *V. cholerae* O1 isolated from Lake Victoria were phylogenetically identical to those causing cholera outbreaks in the African Great Lakes region further illustrates the association between Lakes and cholera [23].

## Limitations

The principal limitation of this study is the weakness of the cholera surveillance system in Tanzania to provide disaggregated data available weekly and at district levels in order to serve as data for action to support the roadmap for cholera control. Moreover, there is a low incidence of cholera in Tanzania based on reported data and this could be attributed to the fact that reporting cholera has negative impacts on tourism and exports of affected countries, leading to underreporting. Furthermore, Dar es Salaam is one on of the main cities experiencing recurrent cholera outbreaks in Tanzania but was not identified as part of the hotspot areas because data were aggregated at regional level and could bias the spatial analysis. Data aggregated at regional levels were not able to detect rainfall as a significant risk factor on cholera incidence. During data curation we observed that only a small proportion of suspected cases are laboratory confirmed and this may affect the consistency in case definitions and identification of priority areas where interventions are needed.

## Supplementary information

**Additional file 1.** Number of cholera reported cases and deaths by region and cholera risk factors.

## Data Availability

The dataset supporting this study has been submitted in Additional file 1.

## Abbreviation

DRC: Democratic Republic of Congo
